# Cross-species analysis uncovers the mitochondrial stress response in the hippocampus as a shared mechanism in mouse early life stress and human depression

**DOI:** 10.1016/j.ynstr.2024.100643

**Published:** 2024-05-14

**Authors:** Bente M. Hofstra, Emmy E. Hoeksema, Martien JH. Kas, Dineke S. Verbeek

**Affiliations:** a Department of Genetics, University of Groningen, University Medical Center Groningen, the Netherlands; b Department of Behavioural Neuroscience, Groningen Institute for Evolutionary Life Sciences, University of Groningen, the Netherlands

**Keywords:** Depression, Early life stress, Hippocampus, Mitochondrial stress response, RNA-Sequencing, Cross-species analysis, Transcriptome, Maternal separation

## Abstract

Depression, or major depressive disorder, poses a significant burden for both individuals and society, affecting approximately 10.8% of the general population. This psychiatric disorder leads to approximately 800,000 deaths per year. A combination of genetic and environmental factors such as early life stress (ELS) increase the risk for development of depression in humans, and a clear role for the hippocampus in the pathophysiology of depression has been shown. Nevertheless, the underlying mechanisms of depression remain poorly understood, resulting in a lack of effective treatments. To better understand the core mechanisms underlying the development of depression, we used a cross-species design to investigate shared hippocampal pathophysiological mechanisms in mouse ELS and human depression. Mice were subjected to ELS by a maternal separation paradigm, followed by RNA sequencing analysis of the adult hippocampal tissue. This identified persistent transcriptional changes linked to mitochondrial stress response pathways, with oxidative phosphorylation and protein folding emerging as the main mechanisms affected by maternal separation. Remarkably, there was a significant overlap between the pathways involved in mitochondrial stress response we observed and publicly available RNAseq data from hippocampal tissue of depressive patients. This cross-species conservation of changes in gene expression of mitochondria-related genes suggests that mitochondrial stress may play a pivotal role in the development of depression. Our findings highlight the potential significance of the hippocampal mitochondrial stress response as a core mechanism underlying the development of depression. Further experimental investigations are required to expand our understanding of these mechanisms.

## 1 Introduction

Depression, or major depressive disorder, places a major burden on both patients and society (World Health Organization, 2019). The psychiatric disorder affects 10.8% of the general population (Lim et al., 2018), leading to 800,000 deaths and up to 1 trillion US dollars in economic burden each year (World Health Organization, 2019). Depression has a major impact on quality of life and often leads to occupational dysfunction, as well as social isolation (Rapaport et al., 2005). Patients experience loss of energy, impaired cognitive function, and anhedonia. The development of depression is influenced by a combination of environmental and genetic factors. As of yet, however, the underlying mechanisms are poorly understood, and this is reflected in the lack of effective treatment options. A better understanding of the mechanisms of disease is urgently needed to improve treatment efficacy and relieve the personal and societal burden.

One of the reasons for our poor understanding of the mechanisms of disease is the inaccessibility of the affected tissue (brain) outside of end-stage disease. In addition, the heterogeneity of the patient population in terms of genetic background, environmental factors, and life experiences makes it extremely challenging to disentangle the causes of disease. The use of experimental animals is a solution to control for these factors, as control of genetic background, environment, and life experiences is achievable in animal models (Homberg et al., 2021). However, the existing paradigms lead to depressive-like symptoms and treatments that are often only efficient in these disease models. For example, substance P antagonists and neuropeptide receptor ligands showed great effectivity in animal studies but did not show the expected results in humans (Belzung, 2014; Herzog et al., 2018; Herpfer and Lieb, 2005; Griebel and Holsboer, 2012). Given these challenges inherent to human postmortem studies and animal models with depressive-like symptoms, we hypothesised that by using a cross-species design that focusses on risk factors and genetic pathways in specific brain regions, we may be able to identify conserved basic mechanisms underlying the development of depression.

One of the known environmental risk factors at play in depression in humans is early life stress (ELS) (LeMoult et al., 2020). ELS, also referred to as childhood trauma, childhood maltreatment, or early life adversity, refers to traumatic events early in life. In humans, this often entails childhood abuse, including emotional, physical, and sexual abuse; childhood neglect, both emotional and physical; and/or other traumatic events such as passing of a parent or guardian. These adverse events in early life have a major impact on the still-developing brain. ELS increases the chance of depression by 2.5, and 46% of depressive patients have a history of ELS (Nelson et al., 2017). Taken together, ELS has been demonstrated to be a clear risk factor for the development of depression (LeMoult et al., 2020; Merrick et al., 2017), and ELS (Hou et al., 2022)is therefore often used as an environmental stimulus to induce depressive-like behaviour in experimental animals (Nakama et al., 2023; Rupasinghe et al., 2022; Schmidt et al., 2011).

In humans, ELS resulted in smaller volumes of subcortical brain areas, including the hippocampus (Frodl et al., 2017), which also displayed aberrant functioning (Lambert et al., 2019). Reduced hippocampal volume is also a robust pathology/hallmark in patients with depression (Campbell et al., 2004; Schmaal et al., 2016; Ho et al., 2022). Importantly, this reduction in volume could already be seen in individuals who experienced ELS prior to their development of depression (Calem et al., 2017). This indicates that the hippocampal shrinkage is not merely a consequence of depression but rather a consequence of ELS. The relevance of the hippocampus in ELS has been illustrated by a combination of studies showing a decrease in hippocampal volume, changes in associative learning in humans, and impairments in synaptic pruning in rodents that resulted in abnormal connectivity (Lambert et al., 2017, 2019; Dayananda et al., 2023).

A possible explanation for the vulnerability of this specific brain region could be its active development during childhood (Khalaf-Nazzal and Francis, 2013; Brunson et al., 2003). Decrease in cognitive performance in depressive patients illustrates the decrease in hippocampal functioning (Bora et al., 2013; MacQueen and Memedovich, 2017). Furthermore, both depression and ELS are associated with aberrant reward processing (Herzberg and Gunnar, 2020; Keren et al., 2018). The hippocampus is thought to be an upstream regulator of this process, possibly by modulating the responsiveness of dopaminergic neurons (Lodge and Grace, 2006). Taken together, these studies illustrate that disruption of hippocampal development early in life has consequences for hippocampal functioning later in life.

In rodents, ELS can be induced through different paradigms such as chronic unpredictable stress, maternal separation (MS), limited bedding and nesting material (LBN), and maternal deprivation (Schmidt et al., 2011; Schuler et al., 2022). ELS via MS as well as LBN is known to induce hippocampal effects such as decreased performance in the Morris water maze and object recognition tasks (Rocha et al., 2021). What is more, a recent meta-analysis by Wang et al. indicated that mice do not typically develop severe depressive-like behaviours after ELS through MS (Wang et al., 2020a). The presence of hippocampal symptoms in absence of depressive-like symptoms further indicates that hippocampal changes might precede the development of depression-like behaviours, as well as underlining the brain-region-specific effects underlying the mechanisms of depression.

To our knowledge, two studies have previously investigated the mouse hippocampal transcriptome after MS (Alberry et al., 2020; Kiser et al., 2019). However, both used a separation period of post-natal day (PND) 1–14 that was previously shown not to result in increased vulnerability to developing depression later in life (Peña et al., 2017). In contrast, MS between PND 10–17 was shown to lead to increased vulnerability to depressive-like symptoms in adulthood (Peña et al., 2017).

Therefore, in the present study, we subjected male CL57 B/L mice to either MS + LBN or standard rearing (SR) between PND 10–17 and conducted RNA sequencing (RNAseq) analysis on hippocampal tissue to investigate putative long-term transcriptional effects of ELS (±10 weeks). We identified differentially expressed genes (DEGs) upon MS treatment, performed pathway enrichment, and predicted protein interactions through the STRING database. We then compared these data to data from a publicly available gene expression dataset originating from postmortem hippocampal tissue of depressive patients (Lanz et al., 2019). This identified shared cross-species genes and pathways that provide new insights into conserved mechanisms underlying the development of mouse ELS and human depression.

## 2 Methods

### 2.1 Animals

Male and female C57BL/6 mice were bred in-house. Mice were housed on a 12hr/12hr light/dark cycle. MS occurred from PND 10–17 (see section *Maternal separation*). After weaning at PND 21, animals were group-housed in groups of 3 with same-sex litter mates in standard polypropylene 2L cages in a temperature-controlled room (21 ± 2 °C) on a 12hr/12hr light/dark cycle. Food and water were available *ad libitum*. After weaning, all cages were outfitted with paper shreds as nesting material, a cardboard tube, and a triangular, red see-through hide. Animals were sacrificed at 10 weeks of age by decapitation. Hippocampi were dissected immediately after sacrifice and snap frozen and stored at −80 °C until further processing.

All procedures conformed to Directive 2010/63/EU of the European Parliament on the protection of animals used for scientific purposes and approved by the national Central Authority for Scientific Procedures on Animals (CCD) and the Institutional Animal Care and Use Committee (IVD) of the University of Groningen.

### 2.2 Maternal separation

Generally, physical and emotional neglect is mimicked by separation of the intact nest from the dam for 1–8 hr per day for several consecutive days. Peña et al. stratified the timing of separation to be optimal between PND 10 and 17, and earlier MS does not seem to induce behavioural effects (Peña et al., 2017). One consequence of the MS paradigm is that dams often increase the level of maternal care upon the return of the litter (Millstein and Holmes, 2007; Orso et al., 2019). LBN can be used to counteract this behaviour because it results in fragmented maternal care (Orso et al., 2019).

**Fig. 1:**
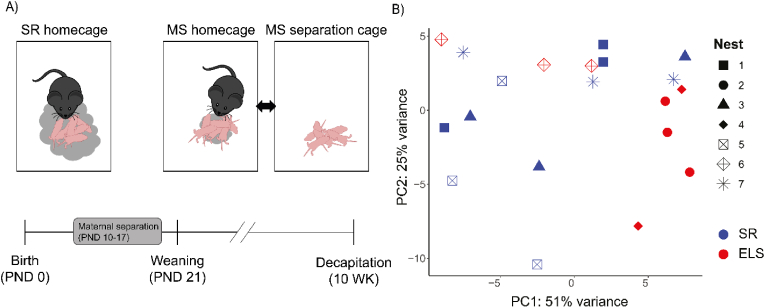
A) Timeline and visualization of the MS paradigm B) PCA analysis of batch effects between different nests. Nests are represented by symbols and rearing conditions are represented by color, red for ELS and blue for SR. (For interpretation of the references to color in this figure legend, the reader is referred to the Web version of this article.)

Within 24 hr after birth, pups were sexed and balanced over dams so that each litter consisted of 3 males and 3 females. Nest health was monitored for weight, temperature, and visual signs of distress. The MS protocol was adapted from Peña et al. (2017). Whole nests were randomly assigned to either the MS or the control group. MS occurred for 4 hr daily from PND 10 to PND 17, and the nests were transferred to a new cage as a whole (Fig. 1A). Dams remained in the home cage and were placed on the opposite side of the room. Cages containing separated nests were placed on a heating pad for 1/3 of the cage to prevent hypothermia. In MS home cages, 2/3 of the nesting material was removed in order to counteract increased maternal care after return of the nests. Bedding of MS nests was restored upon return to the home cage on PND 17.

### 2.3 RNA isolation and sequencing

RNA was extracted using Trizol following the manufacturers protocol (Ambion Inc, USA). Quality control and Poly-A sequencing was performed by GenomeScan (Leiden, the Netherlands) using Illumina NovaSeq6000 sequencing, with a read depth of 20 million 150bp paired-end reads per sample. All samples passed quality control with RQN values > 8.9 and 18s/28s values > 1.1.

### 2.4 Processing sequencing data

Reads were aligned using STAR (v2.7.3a-foss-2018b) with GRCm38.102 as reference genome. Alignment scores exceeded 93% for all samples. Aligned reads were further processed using the FeatureCounts package in RStudio (v1.3.1093). Outlier detection in the form of principal component analysis (PCA) revealed one clear outlier in the data. The outlier was confirmed through hierarchical clustering analysis and removed before any further analysis. Next, DEGs were determined using DESeq2, and the BioMart package was used to annotate gene symbols. The IHW (independent hypothesis weighting) package was used to adjust p values for multiple testing.

### 2.5 Pathway analysis

Pathway analysis was conducted in Metascape using default parameters of the express analysis (Zhou et al., 2019). The software includes annotations from the KEGG (Kanehisa and Goto, 2000), GO (Ashburner et al., 2000) and Reactome (Jassal et al., 2020) databases.

### 2.6 STRING analysis

DEGs were uploaded into Cytoscape (Shannon et al., 2003) (v3.10.0). The protein-protein interaction (PPI) network was constructed through stringApp (Doncheva et al., 2019) (v2.0.1) using the *Mus Musculus* background set and standard parameters. The enrichment function within stringApp was employed to identify biological pathways. The overlap cutoff was set to 0.7, and pathways including >1000 genes were manually removed.

## 3 Results

### 3.1 MS results in persistent hippocampal transcriptional changes

To assess long-term transcriptional changes in mouse hippocampus as a result of MS, we induced ELS by MS in male mice following the protocol described in the Materials and Methods section (Fig. 1A). After 10 weeks, the hippocampi of the respective mice were isolated followed by bulk RNAseq on the hippocampal RNA samples of the MS group (n = 9) and the control SR group (n = 12).

**Fig. 2:**
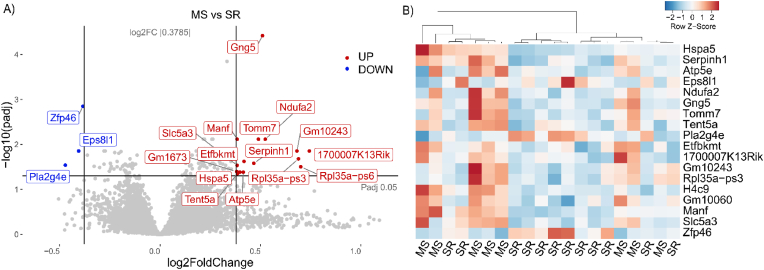
A) Volcano plot of DEGs. Each gene is represented by a dot. Statistical significance is illustrated by the horizontal line, biological relevance is illustrated by the vertical lines. Genes that fulfill both these parameters are represented by blue (downregulated) and red (upregulated) coloring. B) Heatmap of biologically- and significantly relevant DEGs. (For interpretation of the references to color in this figure legend, the reader is referred to the Web version of this article.)

First, the RNAseq data was filtered for existing mouse transcripts, which yielded 55,487 genes as annotated in Ensembl. Second, PCA confirmed the absence of nest effects and did not show a clear clustering of the two groups of mice (Fig. 1B). Third, to identify DEGs at the transcript level, the data for the two groups was further analysed using DESeq2. This identified 126 unique DEGs based on a corrected p value < 0.05, including 83 up-regulated DEGs and 43 downregulated DEGs (Fig. 2A, Supplementary Table 1). Fourth, a 1.3-fold-change (FC) cutoff was applied to identify DEGs of biological significance (Peña et al., 2017; Rowson et al., 2019), with 18 transcripts reaching this threshold (Table 1). Further analysis by hierarchical clustering of the biologically relevant DEGs could separate most MS mice from SR mice, and vice versa (Fig. 2B).

**Table 1:** DEGs of biological relevance.

Mouse gene symbol	Description	Log2 fold change	P value adjusted	Human gene ID	Human gene symbol
*Gng5*	guanine nucleotide binding protein (G protein), gamma 5 [Source:MGI Symbol; Acc:MGI:109164]	0.510	9.13E-05	ENSG00000174021	*GNG5*
*Zfp46*	zinc finger protein 46 [Source:MGI Symbol; Acc:MGI:99192]	−0.385	1.24E-03	ENSG00000125945	*ZNF436*
*Manf*	mesencephalic astrocyte-derived neurotrophic factor [Source:MGI Symbol; Acc:MGI:1922090]	0.384	9.61E-03	ENSG00000145050	*MANF*
*Ndufa2*	NADH:ubiquinone oxidoreductase subunit A2 [Source:MGI Symbol; Acc:MGI:1343103]	0.523	1.06E-02	ENSG00000131495	*NDUFA2*
*Tomm7*	translocase of outer mitochondrial membrane 7 [Source:MGI Symbol; Acc:MGI:1913419]	0.489	1.55E-02	ENSG00000196683	*TOMM7*
*Eps8l1*	EPS8-like 1 [Source:MGI Symbol; Acc:MGI:1914675]	−0.405	1.82E-02	ENSG00000131037	*EPS8L1*
*1700007K13Rik*	RIKEN cDNA 1700007K13 gene [Source:MGI Symbol; Acc:MGI:1916577]	0.743	2.08E-02	ENSG00000160345	*PIERCE1*
*Serpinh1*	serine (or cysteine) peptidase inhibitor, clade H, member 1 [Source:MGI Symbol; Acc:MGI:88283]	0.466	2.42E-02	ENSG00000149257	*SERPINH1*
*Slc5a3*	solute carrier family 5 (inositol transporters), member 3 [Source:MGI Symbol; Acc:MGI:1858226]	0.384	2.56E-02	ENSG00000198743	*SLC5A3*
*Rpl35a-ps3*	ribosomal protein L35A, pseudogene 3 [Source:MGI Symbol; Acc:MGI:3704473]	0.689	2.66E-02		
*Hspa5*	heat shock protein 5 [Source:MGI Symbol; Acc:MGI:95835]	0.397	2.90E-02	ENSG00000044574	*HSPA5*
*H4c9*	H4 clustered histone 9 [Source:MGI Symbol; Acc:MGI:2448432]	0.822	3.29E-02		
*Gm10060*	predicted gene 10060 [Source:MGI Symbol; Acc:MGI:3710638]	0.743	3.38E-02		
*Etfbkmt*	electron transfer flavoprotein beta subunit lysine methyltransferase [Source:MGI Symbol; Acc:MGI:2443575]	0.418	3.39E-02	ENSG00000139160	*ETFBKMT*
*Atp5e*	ATP synthase, H+ transporting, mitochondrial F1 complex, epsilon subunit [Source:MGI Symbol; Acc:MGI:1855697]	0.414	3.62E-02	ENSG00000124172	*ATP5F1E*
*Gm10243*	predicted gene 10243 [Source:MGI Symbol; Acc:MGI:3704266]	0.681	4.39E-02		
*Pla2g4e*	phospholipase A2, group IVE [Source:MGI Symbol; Acc:MGI:1919144]	−0.472	4.39E-02	ENSG00000188089	*PLA2G4E*
*Tent5a*	terminal nucleotidyltransferase 5A [Source:MGI Symbol; Acc:MGI:2670964]	0.385	4.75E-02	ENSG00000112773	*TENT5A*

### 3.2 Enrichment analysis reveals oxidative phosphorylation and protein folding as core mechanisms affected by MS

To identify the pathways underlying the long-term transcriptional effects of MS, we performed an enrichment analysis for all significant DEGs. Transcripts differentially expressed in the MS group were mainly involved in *oxidative phosphorylation* (p value = 1.82x10^−10^), *SRP-dependent cotranslational protein targeting to membrane* (p value = 1.00x10^−6^), and *protein folding* (p value = 6.76x10^−04^) (Table 2, Supplementary Tables 1 and 2). One third of the DEGs with 1.3-FC were involved in oxidative phosphorylation and included *gng5*, *ndufa2*, *tomm7*, *slc5a3*, *hspa5*, and *atp5e*. These genes seem to drive the enrichment of this pathway as they make up 43% of the MS DEGs in this pathway. Genes with a biologically relevant FC made up 40% of the MS group genes involved in response to unfolded protein. These observations substantiate the biological relevance of these pathways, and such marked enrichment was not seen for other pathways.

**Table 2:** Pathway enrichment analysis of all significant DEGs.

GO	Category	Description	Count	%	P value adjusted
mmu00190	KEGG Pathway	Oxidative phosphorylation	14	12.84	1.82E-10
R-MMU-1799339	Reactome Gene Sets	SRP-dependent cotranslational protein targeting to membrane	9	8.26	1.00E-06
GO:0006457	GO Biological Processes	Protein folding	8	7.34	6.76E-04
mmu04932	KEGG Pathway	Non-alcoholic fatty liver disease	7	6.42	4.07E-03
GO:0035176	GO Biological Processes	Social behavior	5	4.59	4.68E-03
GO:0006986	GO Biological Processes	Response to unfolded protein	5	4.59	2.88E-02

**Fig. 3:**
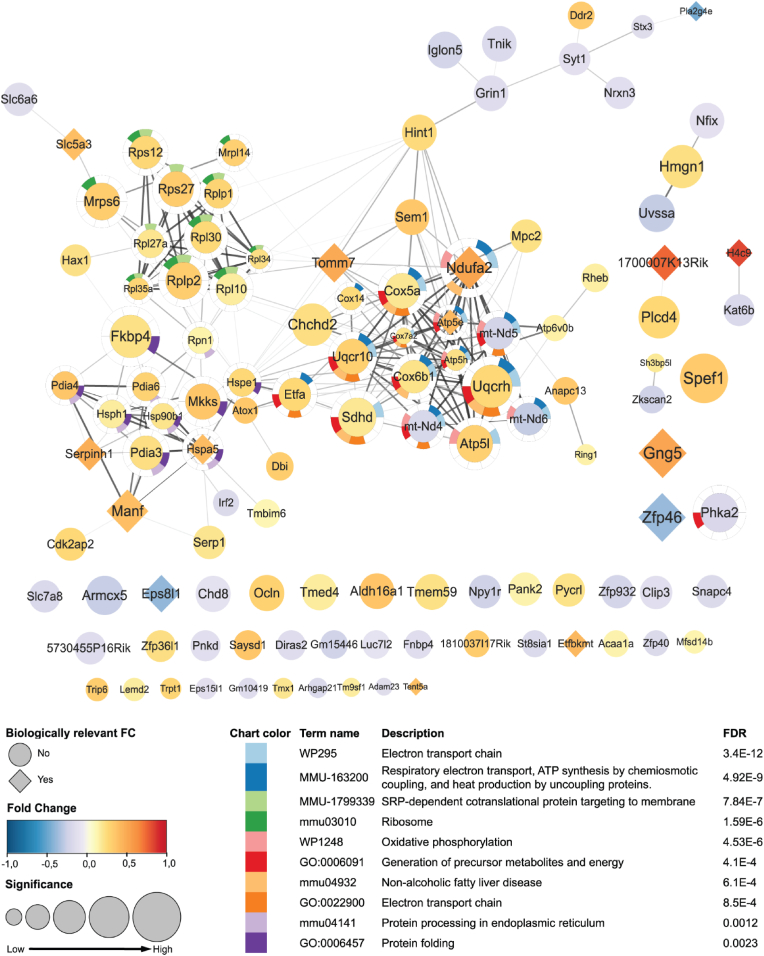
Protein-protein interaction network and STRING analysis of all DEGs. Shapes represent biological relevance, fold change is represented by a blue (down regulated) to red (upregulated) color gradient. Node size increases with increased significance. Genes that are part of one or more enriched pathways are surrounded by a halo, in which each color corresponds to a pathway as presented in the table. (For interpretation of the references to color in this figure legend, the reader is referred to the Web version of this article.)

Notably, the PPI network generated with STRING (Doncheva et al., 2019) based on the proteins translated from the DEGs revealed the enrichment of functionally similar biological pathways, including *the electron transport chain*, *SRP-dependent cotranslational protein targeting to membrane*, and *protein processing in the endoplasmic reticulum*, to be implicated in the long-term effects of MS (Fig. 3).

### 3.3 Mitochondrial stress pathways show cross-species conservation in depression

For the human part of our cross-species analysis, we made use of a publicly available transcriptome dataset for depression from postmortem human hippocampal tissue (Lanz et al., 2019). This work includes postmortem samples from the prefrontal cortex, striatum, and hippocampus among matched tetrads of controls, with subjects diagnosed with schizophrenia, bipolar, or major depressive disorder and control subjects. Only data pertaining hippocampal tissue from depressive patients was extracted from this dataset. Data related to other disorders and brain regions was disregarded. The hippocampal-depression subset comprised of 295 significant DEGs between depressive subjects and healthy controls.

To compare the mouse MS DEGs with the human depression dataset, we converted the 125 significant mouse DEGs into human transcripts, which identified 111 unique transcripts (Supplementary Table 1). When comparing these 111 human DEGs (MS) with the 295 human DEGs (depression), we identified six shared DEGs with opposite FC directionality between the two datasets: *GNG5, TRIP6, ATP5MG, COX5A, COX6B1,* and *UQCRH.* Of these six genes, *GNG5, ATP5MG, COX5A, COX6B1,* and *UQCRH* are involved in *the citric acid (TCA) cycle* and *respiratory electron transport,* among others (Supplementary Table 3).

**Fig. 4:**
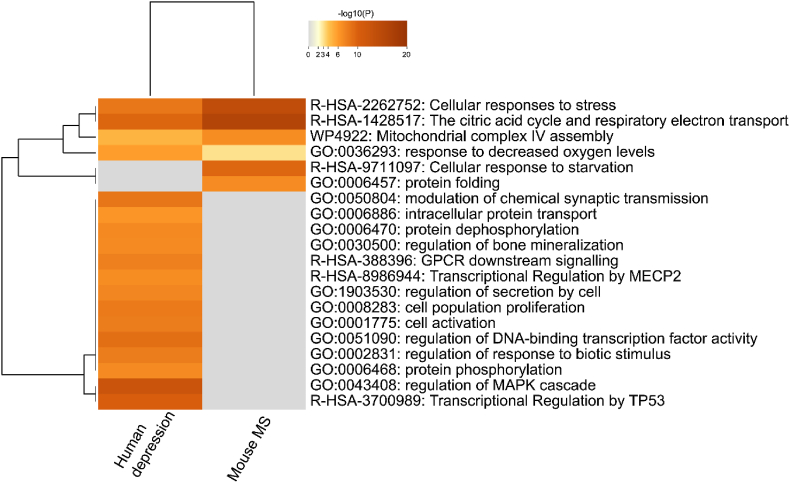
Heatmap of the 20 most significantly enriched pathways for human depression and mouse MS. Significance is represented by a color gradient. (For interpretation of the references to color in this figure legend, the reader is referred to the Web version of this article.)

Upon comparing the enriched biological pathways between the depression dataset and the 111 human orthologs stemming from the MS DEGs, we identified six overlapping core pathways involved in cellular stress and mitochondrial stress responses (Fig. 4). This analysis shows that the core mechanisms underlying the development of depression and ELS are likely involved in the mitochondrial stress response.

## 4 Discussion

In this study we applied a cross-species design using hippocampal mouse ELS and human depression transcriptome data to determine core mechanisms underlying the development of depression. This work has shown that ELS by MS causes long-term transcriptional changes in the mouse hippocampus. Further, pathway analysis revealed that the mitochondrial stress response and protein folding are the pathways most affected by MS in mice. Most importantly, our comparison of the mouse ELS hippocampus data with human data revealed mitochondrial stress responses in the hippocampus as a potentially conserved mechanism related to the development of depression and ELS.

In this work, six genes were identified to be differentially expressed in both mice and human: *GNG5*, *TRIP6*, *ATP5MG*, *COX5A*, *COX6B1*, and *UQCRH*. Four of these genes are directly involved in mitochondrial function as they are part of respiratory chain complexes III (*UQCRH*), IV (*COX5A* and *COX6B1*) and V (*ATP5MG*). Interestingly, these six DEGs showed opposite directionality, with downregulation in humans and upregulation in mice. We hypothesize that this difference in directionality stems from the difference in disease stages. The human samples stem from late stages in which we expect to see downregulation of these genes in correspondence with decreased mitochondrial function as observed in depressive patients (Martins-De-Souza et al., 2012; Abdallah et al., 2014). Our mouse samples were taken at very early stages. Upregulation of these mitochondrial genes in these early stages might be part of a compensatory mechanism in an effort to rescue mitochondrial function.

Mitochondrial function has previously been linked with depression on both the genetic and the functional level (Allen et al., 2018; Rappeneau et al., 2020). Polymorphisms in mitochondrial genes such as *TOMM40*, *ATP6V1B2*, and *MOA* are correlated to depression in humans (reviewed in (Petschner et al., 2018)). Further, 68% of depressive patients present with mtDNA deletions, almost double that of healthy controls (36%) (Gardner et al., 2003). Mutations in genes involved in mtDNA stability in particular are correlated to disease onset as well as treatment outcome (Czarny et al., 2023). mtDNA copy number (mtDNAcn) is often used as a proxy of mitochondrial function (Picard, 2021). However, studies on mtDNAcn levels in depressive patients have yielded inconsistent results, with increases (Tyrka et al., 2016), decreases (Chang et al., 2015), and no differences reported (Lindqvist et al., 2018; He et al., 2014). Other studies reporting on whole proteome analysis on the dorsolateral prefrontal cortex of depressive patients showed differential expression of proteins involved in metabolism and energy (Martins-De-Souza et al., 2012). Furthermore, ATP levels seem to be decreased in these depressive patients compared to healthy controls (Martins-De-Souza et al., 2012). Magnetic resonance spectroscopy also showed a decrease in mitochondrial energy production in depressive patients (Abdallah et al., 2014). Taken together, these studies clearly indicate mitochondrial involvement in depression.

Notably, patients with other neuro(psychiatric) disorders such as bipolar disorder, schizophrenia, and Alzheimer's disease are often comorbid for depressive symptoms (Lyketsos and Olin, 2002; Krynicki et al., 2018). In these diseases mitochondrial involvement has also been reported, in the absence of comorbid depression (Das et al., 2022; Kato, 2017; Weidling and Swerdlow, 2019). This includes mtDNAcn variations, aberrant mitochondrial enzyme activity, and changes in mitophagy, among others (Das et al., 2022; Kato, 2017; Weidling and Swerdlow, 2019). It could thus be speculated that the depressive symptoms in these diseases stem from mitochondrial dysregulation. Combined with our own study findings, we propose that the changes in the mitochondrial stress response are a core mechanism that increases the vulnerability to develop subsequent depressive symptoms.

The involvement of MS DEGs in the process of oxidative phosphorylation, but also in the mitochondrial stress response further point to mitochondrial involvement in the development of depressive symptoms. This aberrant oxidative phosphorylation is in addition to mitochondria with a distorted stress response pathway that are no longer able to safeguard their energy production (Sutandy et al., 2023). This could be very troublesome for neurons as they rely almost exclusively on oxidative phosphorylation to meet their energy demands (Fang et al., 2021; Goyal et al., 2017).

Moreover, changes in mitochondrial function due to ELS are especially detrimental in the hippocampus, since this brain area is still actively developing in early life (Rangaraju et al., 2019). The relevance of this brain area is further illustrated by the significant decrease in its size in depressive patients (Campbell et al., 2004; Schmaal et al., 2016; Ho et al., 2022) and as a result of ELS (Frodl et al., 2017; Calem et al., 2017). Oxidative phosphorylation is very important during the developmental period of the hippocampus. During this period, progenitor cells transition from glycolysis to oxidative phosphorylation as they transition to neurons, increasing the demand for oxidative phosphorylation (Zheng et al., 2016). A failure to meet this increase in demand might result in impaired neurogenesis (Shin et al., 2015; Beckervordersandforth et al., 2017). This increased demand for oxidative phosphorylation in the hippocampus even seems to extend into adulthood, as the hippocampus is the only brain area capable of adult neurogenesis. Adult-born neurons are speculated to fill a unique role as they seem to behave differently from their mature counterparts and have higher firing rates and reduced special tuning (Danielson et al., 2016). Indeed, impairment of neurogenesis leads to increased depressive-like behaviours such as decreased sucrose reference and increased immobility in the forced swim test in mice (Snyder et al., 2011).

Beside the mitochondrial stress response, we also observed a role for the unfolded protein response (UPR) upon MS. The role of the UPR is to maintain proteostasis, and it is mediated through the endoplasmic reticulum (ER) (Liu et al., 2023). The ER and mitochondria are closely connected, with 20% of the mitochondrial surface in direct contact with the ER (Kornmann et al., 2009). Indeed, the UPR is closely related to mitochondrial function. For example, UPR proteins such as ATF4 have been found to regulate the expression of parkin, which is crucial for mitochondrial quality control (Bouman et al., 2011). There is evidence for the involvement of the UPR in depression as its proteins are increased in depressive suicide victims, as well as after chronic restraint stress in rats (Zhang et al., 2014; Bown et al., 2000). Increased expression of UPR genes other than the MS DEGs we found has been reported in postmortem tissue of the prefrontal cortex and in leukocytes of depressive patients (Yoshino and Dwivedi, 2020; Nevell et al., 2014). Of note, this was not observed in the human hippocampal RNA expression dataset used in this study. Changes in expression of UPR genes are very likely to cause dysregulation of proteostasis and ER stress, affecting neuronal vulnerability and functioning (Sivakumar and Krishnan, 2023). However, we are aware that alterations in UPR are not depression-specific, as UPR changes were previously observed in multiple neuropsychiatric disorders (Ochneva et al., 2022).

There was limited overlap between the significant DEGs identified in our study and those identified in earlier experiments. (Alberry et al., 2020; Kiser et al., 2019). Seven of our MS DEGs are shared with those identified in the Kiser study (Kiser et al., 2019), including USP gene *Hsp5a* (also known as *Grp78*) and *Zfp46,* which had biologically relevant FCs but no overlapping DEGs with the Alberry study (Alberry et al., 2020). This lack of overlap is likely due to differences in the study characteristics. Most importantly, the separation period for these two earlier studies occurred almost directly after birth (PND 2), whereas our protocol did not start separation until PND 10. As shown previously, ELS during the early period does not lead to increased vulnerability to depressive symptoms later in life (Wang et al., 2020a; Peña et al., 2017). Since ELS from PND 10–17 does increase this vulnerability, it is likely that ELS leads to different transcriptional changes depending on its timing. In addition, both earlier studies used anaesthesia in their sacrifice protocols, and mitochondrial processes such as oxidative phosphorylation and oxidative stress in the hippocampus are suggested to be affected by anaesthesia (Wang et al., 2020b). Taken together, these procedural differences likely resulted in the limited overlap in DEGs with the present work.

Kos et al. have previously shown that ELS, in the form of LBN, induces transcriptional changes in the hippocampus that are exacerbated after a second stressor (Kos et al., 2023). Pathway analysis revealed overrepresentation of several mitochondria related pathways such as mitochondrial organization, oxidative phosphorylation and mitochondrial transport. Interestingly, this analysis also uncovered protein folding (Kos et al., 2023). Additionally, genes involved in the metabolic process are also differentially regulated after acute adult stress. A single stressful life event in adulthood rapidly upregulates this genset, after which it returns to baseline expression. This indicates that adult stress affects pathways similar to those that are affected by ELS (von Ziegler et al., 2022).

Although our study uncovered 126 significant DEGs, this was not enough to elicit a distinct transcriptomic profile for MS. Despite their identical genetic background, mice show individual differences in response to ELS, which leads to variability within the data (Peña et al., 2017; von Ziegler et al., 2022; Solarz-Andrzejewska et al., 2023). These differences illustrate that stress susceptibility is a combination of genetic and non-genetic factors such as epigenetics, hypothalamo-pituitary-adrenocortical activity, and serotonergic reactivity (Labonté et al., 2012; Hudson et al., 2015; Ebner and Singewald, 2017). ELS is also known to elicit epigenetic regulation such as methylation and histone modifications (de Boer et al., 2017; Czarny et al., 2021; Weaver et al., 2004; Martins de Carvalho et al., 2021). It is possible that MS elicits primarily epigenetic changes that only translate to transcriptional changes after a second stressor. Given the fact that mice subjected to MS do not typically develop behavioural changes (Peña et al., 2017), we are not surprised to not detect major transcriptional changes after MS with the current relatively low sample size. Although transcriptomics alone did not seem sufficient to fully distinguish the two groups, a composite between transcriptomics and other modulators is likely to generate a clearer distinction between MS and control.

In conclusion, our cross-species design study suggests that the mitochondrial stress response in the hippocampus serves as a fundamental shared mechanism between mouse ELS and depression in humans. Similar studies on other experimental models that mimic/exhibit depression-like characteristics and investigations covering the different disease stages of development of depression will contribute to a better understanding of the cross-species mechanisms.

## CRediT authorship contribution statement

**Bente M. Hofstra:** Writing – original draft, Methodology, Investigation, Formal analysis, Data curation, Conceptualization. **Emmy E. Hoeksema:** Visualization, Methodology, Investigation, Formal analysis, Data curation. **Martien JH. Kas:** Writing – review & editing, Supervision, Methodology, Investigation, Funding acquisition, Conceptualization. **Dineke S. Verbeek:** Writing – review & editing, Supervision, Methodology, Investigation, Funding acquisition, Conceptualization.

## Declaration of competing interest

The authors report no conflict of interest.

## Data Availability

Sequence data is available at GEO, accession number GSE254134.
